# Self‐reported sexual assault and dental service use among adolescents: The moderating role of dental pain

**DOI:** 10.1111/eos.70111

**Published:** 2026-06-01

**Authors:** Ândrea Pires Daneris, Letícia Regina Morello Sartori, Marina da Costa Rocha, Flávio Fernando Demarco, Marcos Britto Correa, Sarah Arangurem Karam

**Affiliations:** ^1^ Graduate Program in Dentistry Federal University of Pelotas Pelotas Brazil; ^2^ Graduate Program in Epidemiology Federal University of Pelotas Pelotas Brazil; ^3^ Graduate Program in Health in the Vital Cycle Catholic University of Pelotas Pelotas Brazil

**Keywords:** adolescent, child sexual abuse, dental health services, observational study, oral health

## Abstract

This study investigated the association between self‐reported sexual assault and dental service utilization among Brazilian adolescents (13–17‐yr), considering sex differences and the moderating role of dental pain. Cross‐sectional analyses using data from the 2019 Brazilian National School Health Survey were performed with stata 18.5, applying survey weights. The outcome was dental service use in the previous year, and the exposure was lifetime self‐reported sexual assault. A total of 124,106 adolescents were included; 50.4% were female and 66.3% aged 13–15‐yr. Overall, 66.7% reported dental visits in the previous year, 18.6% reported dental pain, and 6.2% reported sexual abuse. Adolescents with a history of sexual assault showed lower prevalence of dental service use compared with their non‐abused peers (prevalence ratio [PR] = 0.96; 95% confidence interval [CI]: 0.92–1.00), particularly among girls (PR = 0.94; 95% CI: 0.89–0.98). Dental pain was associated with a modest increase in dental service utilization across all groups, with no evidence of effect modification. These findings suggest that sexual abuse is linked to reduced engagement with dental services during adolescence, especially among females, although the magnitude of this association is modest. The results emphasize the importance of trauma‐informed and intersectoral strategies to improve access to oral healthcare for vulnerable populations.

## INTRODUCTION

Sexual violence against children encompasses any completed or attempted, intentional, and nonconsensual act of a sexual nature that occurs before the age of 18, with the potential to cause physical or psychological harm or suffering [1]. Recognized as a widespread and urgent major public health issue, it affects millions worldwide [2, 3]. A recent systematic review estimated the global prevalence of sexual violence against children at 18.9% among females and 14.8% among males [3]. Severe forms of abuse, such as rape and sexual assault, remain worryingly high, affecting around one in every eight girls or women and one in every eleven boys or men [2]. Notably, most first incidents occur during adolescence, particularly between the ages of 12 and 18, highlighting this developmental period as one of increased vulnerability [3, 4]. In Brazil, this pattern is reflected, with adolescents accounting for a substantial proportion of victims [4]. However, underreporting is high, with estimates suggesting that only around 8% of cases of sexual violence against children and adolescents are reported to the authorities [5].

Beyond its high prevalence, sexual violence has profound and lasting effects on the physical, emotional, and psychological development and well‐being of children and adolescents over the life course [6, 7]. In the field of oral health, specific challenges emerge for survivors [8, 9, 10]. Studies have identified elements within the dental environment—such as the lack of control, the sensation of being restrained due to the dental chair position, the invasion of personal space, and anticipated pain—that may act as trauma triggers, evoking past experiences and contributing to revictimization and trauma‐related responses [8, 10, 11, 12]. These responses can lead to heightened anxiety even before the consultation, avoidant behavior, missed appointments, and increased emotional distress [10, 12]. Those who suffered sexual violence also report persistent oral health symptoms, including pain and unmet treatment needs, and discomfort during hygiene routines, often due to sensitivities related to dental products or sensations in the mouth [9].

Consistently, quantitative evidence supports the association between the history of sexual abuse in childhood and high levels of dental fear among both adults [11, 13] and adolescents [14]. This fear can create a so‐called vicious cycle: Individuals avoid dental visits, which, in turn, worsen their oral health and reinforce their dental anxiety [15]. Despite this, few studies have investigated the direct impact of childhood sexual abuse on later dental service utilization, with inconsistent findings [16, 17, 18]. Among adolescents, the impact of childhood sexual abuse on the use of dental services remains underexplored. One study conducted in Nigeria found no statistically significant association, which may be partly explained by the low prevalence of use of dental services in the previous 12 months within the sample (1.2%) [14].

Given this context, certain factors may significantly influence how individuals who have experienced childhood sexual abuse engage with oral health services. Among these, dental pain stands out as both a common driver for seeking care [19] and a contributing factor for dental fear, a psychological barrier frequently reported [11, 13, 14, 20]. Although dental fear and anxiety, and trauma‐related distress may lead to avoidance of dental visits, especially for preventive care, acute or intense pain may lead to this population overcoming the psychological barriers [17]. Thus, dental pain may act as a modifying factor in this relationship, potentially leading to different patterns of service use when present. However, this remains an understudied area, mainly considering adolescents. In all cases, the consequences for oral and emotional health are substantial, contributing to delayed care, worsening oral conditions, and reduced quality of life [9, 21].

Therefore, the present study aimed to assess (1) whether the experience of sexual assault is associated with the use of dental services among Brazilian adolescents, (2) whether dental pain moderates this association, and (3) whether these associations differ between sexes. It is hypothesized that there is an association between self‐reported sexual assault and dental service use, with less use of these services by adolescents with a history of sexual violence; moreover, it is anticipated that dental pain may modify this association, such that the effect of dental pain on service utilization differs according to sexual assault history. Finally, it is expected that these associations may vary by sex, with stronger associations among girls, given known sex differences in healthcare use and trauma‐related responses among males and females [18]. By better understanding the needs of this population, we hope to contribute to the development of more effective intervention strategies that promote equity in access to dental care and improve the quality of life for individuals who experienced child sexual abuse.

## MATERIAL AND METHODS

This study was reported in accordance with the Strengthening the Reporting of Observational Studies in Epidemiology (STROBE) Statement [22].

This observational cross‐sectional study used publicly available data from the 2019 Brazilian National Survey of School Health (2019 PeNSE, *acronym in Portuguese*). The 2019 PeNSE was approved by the National Research Ethics Committee of the Brazilian National Health Council (report no. 3.249.268, issued on April 8, 2019). All participating students provided informed consent, with authorization obtained from their guardians. No incentives, monetary or otherwise, were offered to students or their guardians for participation.

The PeNSE study has had four cross‐sectional waves; the most recent was conducted in 2019. The 2019 PeNSE was a nationwide school‐based survey conducted by the Brazilian Institute of Geography and Statistics in partnership with the Ministry of Health and the Ministry of Education [23]. In Brazil, around 99% of people aged 6–14‐yr and 90% of adolescents aged 15–17‐yr attended school in 2019 [24]. The main objective of the PeNSE study is to monitor and supply up‐to‐date data on the prevalence and distribution of risk and protective health‐related factors among Brazilian adolescents nationwide [23].

In the 2019 PeNSE study, a sample of students aged 13–17‐yr enrolled and in regular attendance at public and private schools in the five Brazilian regions was approached. A two‐stage conglomerate sampling involved selecting schools first, then classes of students. Sampling strata were developed considering the Brazilian federative units and the administrative dependence of the schools (public or private). Subsequently, allocation strata were created based on class size ranges. Classes were then randomly selected within the sampling strata. All the students present on the questionnaire administration day in the randomly selected classes were invited to take part in the study [23].

There were expected to be a total of 4361 schools, 6803 classes, and 187,957 students participating. In the end, a total of 4242 schools, 6612 classes, and 159,245 valid questionnaires took part in the 2019 PeNSE [23]. A total of 119 schools were excluded from the final sample because they were deactivated, did not have eligible classes, or were discarded after collection due to the classes not meeting the criteria for class utilization. Data collection was performed from April to September 2019, through a self‐administered electronic questionnaire in a mobile collection device. Additional methodological information about the 2019 PeNSE study can be found elsewhere [23].

The outcome variable analyzed was the use of dental services in the past year, collected through the question: “In the past 12 months, how many times did you go to the dentist?” The question could have been answered with “none,” “once,” “twice,” or “three times or more.” Responses were dichotomized into “no use in the last 12 months” and “use in the last 12 months.” The moderating variable was self‐reported dental pain in the past 6 months, except for toothaches caused by wearing braces. This item could be answered as “No” or “Yes.” Both items are grounded in methodological recommendations from the World Health Organization for oral health surveys, which propose time intervals that are short enough to estimate treatment needs and service use while minimizing recall bias [25].

The exposure variable was lifetime self‐reported sexual assault, collected from the question: “Has anyone ever threatened, intimidated, or forced you to have sexual intercourse or any other sexual act against your will?” This item could be answered as “No” or “Yes.” This item has been used to determine the prevalence of lifetime exposure to sexual violence, having been applied in the 2015 and 2019 PENSE surveys, as previously evidenced [26, 27]. Adolescents who reported sexual violence were also asked about the age at first abuse and the perpetrator of the abuse through the item “How old were you when someone threatened, intimidated, or forced you to have sexual intercourse or any other sexual act against your will for the first time?”—categorized in ≤12‐yr of age, 13–15‐yr of age, and 16–17‐yr of age, and “Who did this?”—categorized as intimate partner, friend, parents, other family members, stranger, and another person [27].

**FIGURE 1:**
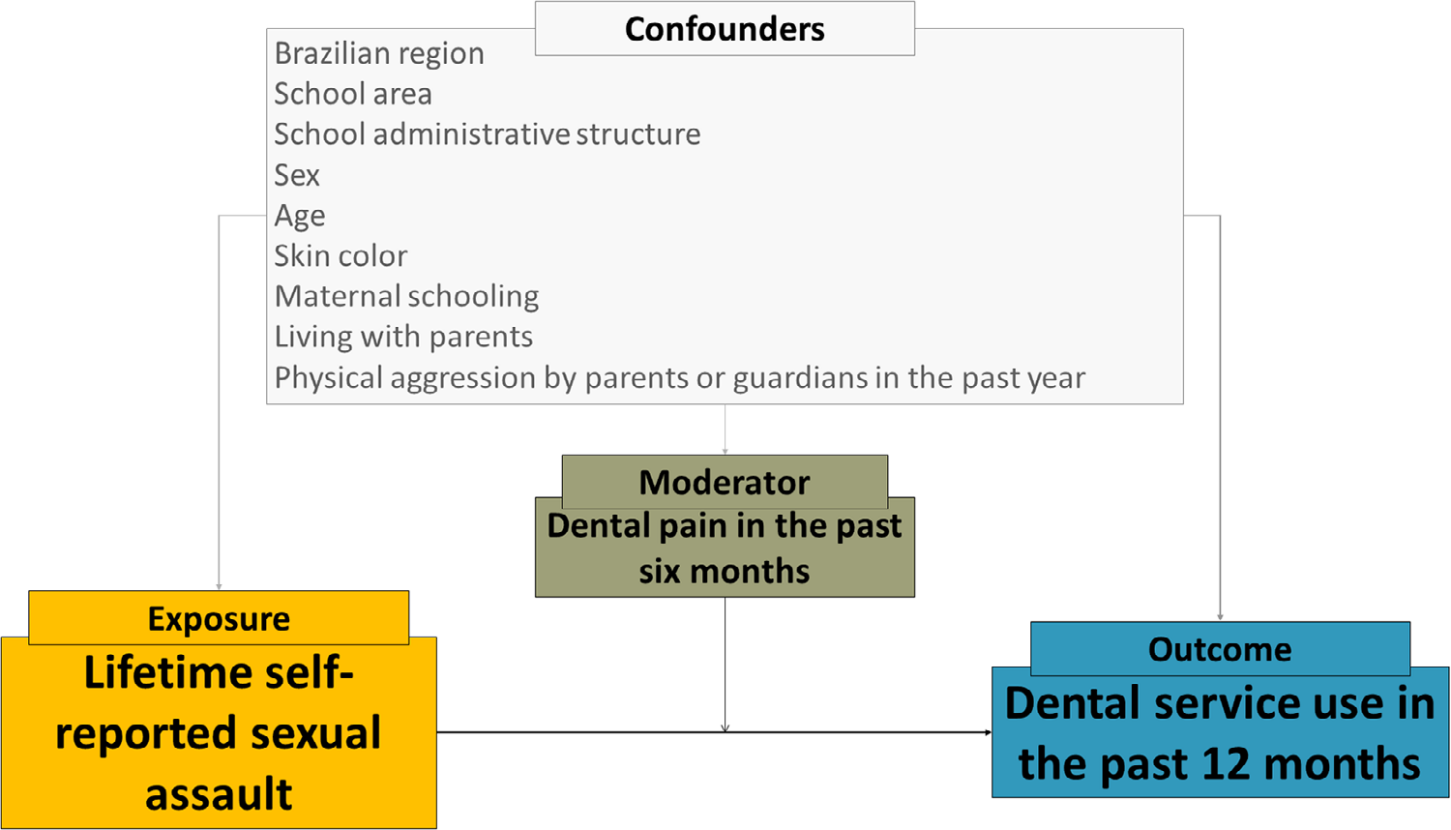
Directed acyclic graph (DAG) for exploring the association between self‐reported sexual assault and dental service use.

To control for potential bias in analysis, we considered the following variables as confounders: Brazilian region (South, Southeast, Center‐West, Northeast, and North); school location (urban or rural); school administrative structure (public or private); sex (male or female); skin color (White, Black, Brown, Yellow, and Indigenous); age (13–15‐ and 16–17‐yr); maternal education (no schooling or incomplete primary school, complete primary school, complete secondary school, and complete higher education); family structure (living with neither parent, living with only father or mother, living with both); and having experienced any physical aggression by caregivers in the last year (no, yes). To support our analysis, a directed acyclic graph was developed to illustrate the relationships between variables a priori (Figure 1).

To be included in this study, questionnaires had to be completed by individuals aged between 13 and 17‐yr‐old; participants below this age were not eligible to answer items on sexual violence. Responses coded as system missing (questionnaire abandonment) or item nonresponse (no response) were treated as missing values.

Weighted analyses accounted for the complex survey design, using the *svy* command in the stata 18.5 software (StataCorp LLC). The relative frequencies for the variables of interest were initially estimated. Bivariate associations between the exposure variable, moderator, and confounders with the use of dental services in the past year were tested using Pearson's chi‐square test. Following, survey‐weighted Poisson regression models with a log link function were fitted sequentially with increasing levels of adjustment. First, crude models were estimated. Next, a minimally adjusted model, including age, sex, and skin color, was fitted. Finally, fully adjusted models were estimated, additionally including all remaining covariates identified a priori as potential confounders. Prevalence ratios (PRs) and 95% confidence intervals (95% CIs) were obtained.

To formally assess effect modification between sexual assault history and dental pain on dental service use, we fitted a multivariable Poisson regression model with robust variance, including the main effects of sexual assault and dental pain, as well as their multiplicative interaction term (sexual assault × dental pain). Statistical evidence of interaction was evaluated using the Wald test for the interaction coefficient, with results presented as PRs and 95% CIs on the multiplicative scale. To explore potential differences by sex, all analyses, including interaction models, were conducted independently for males and females. All regression models were estimated using the same procedures as in the main analysis.

Missing data were handled according to their proportion across variables. For the outcome, exposure, and moderator, the proportion of missing values was low (below 2%); therefore, the main analyses were conducted using complete case analysis (casewise deletion). For covariates with higher proportions of missing data, sensitivity analyses were performed by re‐estimating the fully adjusted models after excluding the respective confounding variable.

Additional sensitivity analyses were conducted to evaluate the robustness of the findings. First, the potential impact of missing responses in the lifetime sexual assault variable was assessed through descriptive comparisons of dental service use across all response categories, including item nonresponse and system‐missing cases, as well as through regression models using alternative exposure coding, including a conservative worst case scenario in which all missing responses were classified as having experienced sexual assault. Second, the association between lifetime sexual assault and dental service use was re‐estimated using an alternative operationalization of the exposure based on the age at first sexual assault, categorized as no history of sexual assault, first abuse at 13–15‐yr of age, and first abuse at ≤12‐yr of age. A significance level of 5% was considered in all analyses.

## RESULTS

Of the 159,245 valid entries, 157,777 (99.1%) students provided valid responses for the dental service use item. Among them, 25,504 (16.2%) entries were excluded because they corresponded to individuals under 13‐yr‐old, and 8167 students (5.2%) were excluded for being 18‐yr or older at the time of data collection, which fell outside the 2019 PeNSE sampling criteria. Therefore, a total of 124,106 (78.6%) valid questionnaires filled with valid responses for the outcome were analyzed in this study. Descriptive and inferential analyses were restricted to these observations.

Table 1 shows the descriptive analysis of the sample according to the variables considered in this study. It can be noted that the majority were enrolled in public schools and schools located in urban areas (Table 1). Additionally, 50.4% were female, 66.3% were aged 13–15‐yr, and 57% self‐reported brown or black skin color (Table 1). Approximately 54% of the sample reported maternal schooling of completed secondary school or higher, 55.5% reported living with both parents, and 21.5% reported having suffered some physical aggression by a parent or guardian (Table 1). Dental pain in the past 6 months was reported by 18.6% of adolescents. As also shown in Table 1, the prevalence of use of dental services in the past year was greater in adolescents who lived in the South, studied in a private school, were females, Whites, with mothers with completed higher education, who lived with both their father and mother, and had dental pain in the last 6 months, compared to their counterparts.

**TABLE 1:** Descriptive statistics and associations between interest variables and dental service use in the past year: relative frequencies (%) and 95% confidence intervals (95% CI).

		Use of dental service in the past year		
		No	Yes	
Variables/Categories	Total (%)	% (95% CI)	% (95% CI)	*p‐*value
**Brazilian region (124,106)**				<0.001
North	10.4	37.0 (35.3–38.6)	63.0 (61.4–64.6)	
Northeast	28.4	36.8 (35.7–38.0)	63.1 (62.0–64.3)	
Southeast	39.7	31.5 (30.2–32.8)	68.5 (67.2–69.8)	
South	13.5	28.3 (26.9–29.7)	71.7 (70.3–73.1)	
Centre‐West	8.0	33.0 (31.5–34.5)	67.0 (65.4–68.5)	
**School area (124,106)**				0.003
Urban	92.9	33.0 (32.3–33.7)	67.0 (66.3–67.7)	
Rural	7.1	37.2 (34.4–40.0)	62.8 (60.0–65.6)	
**School administrative structure (124,106)**				<0.001
Public	85.8	35.4 (34.6–36.2)	64.6 (63.8–65.4)	
Private	14.2	20.4 (19.6–21.3)	79.6 (78.7–80.4)	
**Sex (123,819)**				<0.001
Male	49.6	35.2 (34.2–36.2)	64.8 (63.8–65.8)	
Female	50.4	31.4 (30.6–32.2)	68.6 (67.7–69.4)	
**Age (124,106)**				0.524
13–15‐yr‐old	66.3	33.4 (32.6–34.3)	66.6 (65.7–67.4)	
16–17‐yr‐old	33.7	33.0 (32.0–34.0)	67.0 (66.0–68.0)	
**Skin color (121,623)**				<0.001
White	36.1	28.9 (27.9–29.9)	71.1 (70.1–72.1)	
Black	13.6	38.2 (36.5–39.8)	61.8 (60.1–63.5)	
Brown	43.5	35.3 (34.3–36.2)	64.7 (63.8–65.7)	
Yellow	3.8	33.5 (28.5–38.9)	66.5 (61.1–71.5)	
Indigenous	3.0	35.3 (31.9–38.9)	64.7 (61.1–68.1)	
**Maternal schooling (104,549)**				<0.001
No schooling or incomplete primary school	28.7	38.3 (37.0–39.6)	61.7 (60.3–63.0)	
Primary school	17.2	34.0 (32.3–35.6)	66.0 (64.4–67.6)	
Secondary school	32.6	31.8 (30.7–32.8)	68.2 (67.2–69.3)	
Higher education	21.5	22.8 (21.6–24.1)	77.2 (75.9–78.4)	
**Living with parents (124,006)**				<0.001
Neither mother nor father	6.9	39.1 (36.8–41.3)	60.9 (58.7–63.2)	
Mother or father only	37.6	35.8 (34.8–36.8)	64.2 (63.2–65.2)	
Mother and father	55.5	30.9 (30.0–31.7)	69.1 (68.3–69.9)	
**Physical aggression by parents or guardians in the past year (123,398)**				0.189
No	78.5	33.5 (32.8–34.3)	66.5 (65.7–67.2)	
Yes	21.5	32.5 (31.1–33.9)	67.5 (66.1–68.9)	
**Sexual assault (123,322)**				0.002
No	93.8	33.1 (32.4–33.8)	66.9 (66.2–67.6)	
Yes	6.2	36.8 (34.5–39.2)	63.2 (60.8–65.5)	
**Dental pain in the past 6 months (123,959)**				0.034
No	81.4	33.6 (32.9–34.4)	66.4 (65.6–67.1)	
Yes	18.6	31.9 (30.4–33.4)	68.1 (66.6–69.6)	
**Total (124,106)**	–	33.3 (32.6–33.9)	66.7 (66.1–67.4)	–

*Note*: Each variable included in this table indicates the number of valid observations (unweighted *n*). Weighted percentages account for the survey design. Small variations in n reflect item‐specific missing data.

One‐third of the sample reported not having visited the dentist in the preceding 12 months (Table 1). A prevalence of 6.2% of sexual assaults was found (Table 1). As can be seen in Table S1, in the total sample and within both sexes, 54% or more of adolescents were 12‐yr‐old or younger at the time of first abuse; among girls, 39% reported that their first experience of sexual abuse occurred between the ages of 13 and 15. Regarding the perpetrator, approximately 33% of cases in the total sample were perpetrated by a family member, and 25.5% by an intimate partner (Table S1). More than 30% of boys identified an intimate partner or friend as the perpetrator, whereas girls reported abuse mainly by intimate partners, other family members, and strangers (Table S1).

Table 2 presents the crude and adjusted associations between history of sexual assault and dental service use. In the crude model, adolescents who reported sexual assault experience presented a 6% lower prevalence of using dental services in the past year compared to those who did not report such an experience (PR = 0.94; 95% CI: 0.90–0.98). After adjustment, the magnitude of the association remained similar, with only slight attenuation in the fully adjusted model. In sex‐stratified analyses, no association was observed for males. Among females, those who had experienced abuse showed a 6% lower likelihood of using dental services in the fully adjusted model (PR = 0.94; 95% CI: 0.89–0.98) (Table 2). Table S2 presents models considering the age at first sexual assault; adolescents who experienced abuse up to 12‐yr‐old had a lower probability of dental service use in the past year compared to those without a history of abuse; an association was not observed among those who experienced abuse for the first time during adolescence.

**TABLE 2:** Crude and adjusted associations between adolescents’ history of sexual assault and use of dental service in the past year: total sample and sex‐stratified analyses.

	Use of dental services in the past year					
Variables/Categories	_Crude_PR (95% CI)	*p*‐value	_Adj_PR^1^ (95% CI)	*p*‐value	_Adj_PR^2^ (95% CI)	*p*‐value
**Sexual assault**		0.003		0.001		0.051
No	1		1		1	
Yes	0.94 (0.90–0.98)		0.93 (0.90–0.97)		0.96 (0.92–1.00)	
Unweighted n	123,322		120,602		101,451	
**Sex stratified**						
**Male**						
**Sexual assault**		0.374		0.518		0.823
No	1		1		1	
Yes	0.96 (0.89–1.04)		0.97 (0.90–1.04)		1.00 (0.93–1.08)	
Unweighted n	60,379		59,140		48,640	
**Female**						
**Sexual assault**		<0.001		<0.001		0.010
No	1		1		1	
Yes	0.92 (0.88–0.96)		0.92 (0.88–0.96)		0.94 (0.89–0.98)	
Unweighted n	62,657		61,462		52,811	

*Note*: Analytical sample sizes (unweighted *n*) vary across models due to item‐specific missing data and complete case analysis. Weighted estimates account for the complex survey design. Adjusted prevalence ratios (_Adj_PR^1^) for sex, age, and skin color. Adjusted prevalence ratio (_Adj_PR^2^) for Brazilian region, school area, school administrative structure, sex, age, skin color, maternal schooling, living with parents, and physical aggression by parents or guardians in the past year. In sex‐stratified models, the sex variable was not included in the adjustment as stratification already accounts for its confounding role.

Abbreviations: 95% CI, 95% confidence intervals; PR, prevalence ratio.

Sensitivity analyses, excluding maternal schooling, which had 15.8% missing data, yielded estimates with similar direction and magnitude (Table S3). Additional sensitivity analyses addressing missing responses in the lifetime sexual assault variable indicated that dental service use was similar across item nonresponse and system‐missing categories compared with those reporting no history of sexual assault (Table S4). Regression models using alternative exposure coding, including explicit missing categories and a conservative worst case scenario, yielded estimates consistent with the main analyses, with comparable attenuation of the association in fully adjusted models (Table S5).

Table 3 presents the joint association between the history of sexual assault, dental pain, and the use of dental services in the past year. The reference category was adolescents with a history of sexual abuse and no dental pain. Across both crude and adjusted models, the estimated PRs were similar between exposure groups. Among adolescents with a history of sexual assault, the presence of dental pain contributed to a small increase in the prevalence of dental service use (adjPR^2^ = 1.02; 95% CI: 0.95–1.11). A comparable pattern was observed among adolescents without a history of sexual assault but who reported dental pain that had a 7% higher prevalence of using dental services in the past year (adjPR^2^ = 1.07; 95% CI: 1.01–1.12). These estimates indicate that dental pain is associated with a modest increase in dental service utilization, with comparable magnitudes across groups. For females, similar pattern was found (Table 3).

**TABLE 3:** Crude and adjusted associations between the interaction of adolescents’ history of sexual assault and dental pain in the past 6 months and use of dental services in the past year: total sample and sex‐stratified analyses.

	Use of dental services in the past year					
Variables/Categories	_Crude_PR (95% CI)	*p*‐value	_Adj_PR^1^ (95% CI)	*p*‐value	_Adj_PR^2^ (95% CI)	*p*‐value
**Sexual assault **×** dental pain (past 6 months)**						
Assaulted × no dental pain	1		1		1	
Assaulted × dental pain	1.05 (0.97–1.13)	0.165	1.05 (0.98–1.13)	0.165	1.02 (0.95–1.11)	0.485
No assault × no dental pain	1.07 (1.02–1.12)	0.003	1.08 (1.03–1.13)	0.001	1.04 (0.99–1.09)	0.060
No assault × dental pain	1.10 (1.04–1.16)	0.001	1.10 (1.04–1.16)	<0.001	1.07 (1.01–1.12)	0.014
Unweighted n	*123,220*		*120,503*		*101,386*	
**Sex stratified**						
**Male**						
**Sexual assault **×** dental pain (past 6 months)**						
Assaulted × no dental pain	1		1		1	
Assaulted × dental pain	1.09 (0.94–1.27)	0.210	1.09 (0.94–1.26)	0.224	1.06 (0.91–1.22)	0.423
No assault × no dental pain	1.06 (0.96–1.17)	0.215	1.05 (0.95–1.15)	0.298	1.00 (0.92–1.10)	0.859
No assault × dental pain	1.09 (0.98–1.21)	0.097	1.08 (0.97–1.19)	0.139	1.03 (0.93–1.12)	0.541
Unweighted n	*60,303*		*59,066*		*48,592*	
**Female**						
**Sexual assault **×** dental pain (past 6 months)**						
Assaulted × no dental pain	1		1		1	
Assaulted × dental pain	1.02 (0.93–1.12)	0.568	1.03 (0.94–1.12)	0.503	1.01 (0.92–1.11)	0.803
No assault × no dental pain	1.08 (1.03–1.14)	0.001	1.09 (1.03–1.15)	0.001	1.05 (1.00–1.11)	0.034
No assault × dental pain	1.11 (1.04–1.17)	<0.001	1.11 (1.04–1.18)	<0.001	1.08 (1.01–1.15)	0.011
Unweighted n	*62,631*		*61,437*		*52,794*	

*Note*: Analytical sample sizes (unweighted *n*) vary across models due to item‐specific missing data and complete case analysis. Weighted estimates account for the complex survey design. Adjusted prevalence ratio (_Adj_PR^1^) for sex, age, and skin color. Adjusted prevalence ratio (_Adj_PR^2^) for Brazilian region, school area, school administrative structure, sex, age, skin color, maternal schooling, living with parents, and physical aggression by parents or guardians in the past year. In sex‐stratified models, the sex variable was not included in the adjustment as stratification already accounts for its confounding role.

Abbreviations: 95% CI, 95% confidence intervals; PR, prevalence ratio.

A formal test for interaction between sexual assault and dental pain showed no evidence of effect modification on the multiplicative scale (PR = 0.98; 95% CI: 0.91–1.05; *p* = 0.520). Similar results were observed in sex‐stratified analyses for males (PR = 0.94; 95% CI: 0.81–1.08; *p* = 0.378) and females (PR = 0.99; 95% CI: 0.90–1.09; *p* = 0.895), indicating that the association between dental pain and dental service use was comparable across adolescents with and without a history of sexual assault.

Consistent with these findings, marginal predicted probabilities (Table 4) showed small increases in dental service use associated with dental pain across exposure groups. Overall, adolescents with dental pain had slightly higher probabilities of using dental services, regardless of sexual assault history, and no clear differences in this pattern were observed between exposed and unexposed groups or between males and females (Table 4).

**TABLE 4:** Marginal predicted probabilities of dental service use according to sexual assault history and dental pain, from fully adjusted Poisson regression models.

	Use of dental services in the past year
Variables/Categories	Predicted probability (95% CI)
**Sexual assault **×** dental pain (past 6 months)**	
Assaulted × no dental pain	0.64 (0.61–0.67)
Assaulted × dental pain	0.66 (0.62–0.71)
No assault ×no dental pain	0.67 (0.66–0.68)
No assault × dental pain	0.69 (0.67–0.70)
**Sex stratified**	
**Male**	
**Sexual assault **×** dental pain (past 6 months)**	
Assaulted × no dental pain	0.65 (0.59–0.71)
Assaulted × dental pain	0.69 (0.60–0.78)
No assault × no dental pain	0.65 (0.64–0.67)
No assault × dental pain	0.67 (0.65–0.69)
**Female**	
**Sexual assault **×** dental pain (past 6 months)**	
Assaulted × no dental pain	0.65 (0.62–0.68)
Assaulted × dental pain	0.66 (0.60–0.71)
No assault × no dental pain	0.69 (0.68–0.70)
No assault × dental pain	0.70 (0.68–0.73)

*Note*: Models are adjusted for Brazilian region, school area, school administrative structure, sex, age, skin color, maternal schooling, living with parents, and physical aggression by parents or guardians in the past year. In sex‐stratified models, the sex variable was not included in the adjustment as stratification already accounts for its confounding role.

## DISCUSSION

The findings of this study provide important insights into the association between a history of sexual assault and dental service use among Brazilian adolescents. Adolescents who reported sexual assault showed a slightly lower prevalence of dental service use in crude analyses; after adjustment, the magnitude of this association remained similar, although with some attenuation. These results provided support for the study's first hypothesis. Dental pain in the past 6 months was associated with a modest increase in dental service use across all exposure groups. Adolescents with and without a history of sexual assault showed comparable increases in service use in the presence of pain. Consistent with this pattern, no evidence of effect modification was observed in the formal interaction test, indicating that the association between dental pain and dental service use does not differ according to sexual assault history. Notably, there were some distinct findings between the sexes. Among females, associations were observed and appeared slightly stronger than in the overall sample, whereas among males, no clear associations were observed. These findings support the hypothesis that associations may differ by sex, but differences between sexes should be interpreted with caution.

To contextualize and properly interpret the findings, some limitations should be acknowledged. Firstly, the cross‐sectional design limits the causal inference, preventing conclusions about the temporal sequence between variables. To partially address this limitation, a sensitivity analysis considering the age at first abuse was conducted. This analysis showed that adolescents who experienced sexual assault before the age of 12 were less likely to use dental services in the previous year, whereas no association was observed among those who experienced abuse during adolescence. Nevertheless, exposure may also affect confounders and dental pain depending on the timing of abuse, reinforcing the need for longitudinal studies to clarify causal pathways. Second, disclosure of sexual assault is a sensitive issue, and underreporting is expected; however, the proportion of missing data for the exposure variable was very low. Sensitivity analyses using alternative exposure coding, including a conservative worst case scenario, yielded associations in similar magnitude and direction as the main analyses. It suggests a limited risk of differential bias related to exposure nonresponse and indicates that this attenuation is unlikely to be driven solely by missing or misclassified exposure data. Similarly, recall and social desirability biases may affect the self‐reported data on dental service use and dental pain. Nonetheless, the data were collected individually, anonymously, and only for people over the age of 13, trying to minimize the misreporting. Finally, due to limitations in the questionnaire, the dental service use item does not capture the nature of visits (e.g., preventive, dental treatment without pain, and dental treatment with pain), and it was not asked of participants, which could have provided deeper insights into the patterns of associations observed; moreover, other relevant psychosocial variables were not collected.

Notwithstanding the study's limitations, the findings contribute meaningfully to understanding the association between experiences of sexual assault and dental service utilization, considering a probabilistic sample, with good generalizability to the Brazilian adolescents aged 13–17. The slightly lower use of dental services among adolescents with a history of sexual assault is consistent with previous studies highlighting potential barriers to healthcare engagement among individuals exposed to trauma [9, 12]. After adjustment, the magnitude of this association remained similar, suggesting that this relationship is complex and likely influenced by multiple factors. A similar result was observed in a Nigerian adolescent sample, where no association was found between sexual violence and preventive dental service use, although an association with dental fear was reported [14]. In the present study, the attenuation of the association after adjustment may also reflect changes in the analytic sample due to missing data on maternal schooling. In the present study, the lack of association in the adjusted model may also reflect the complexity of underlying mechanisms. Psychological factors, such as dental fear or trauma‐related avoidance, which are frequently reported by those who faced abuse [10, 11, 12], mediate this relationship—yet these constructs were not directly measured in the PeNSE 2019 study. Future research employing formal mediation analyses is needed to better elucidate these pathways and guide targeted strategies to improve access to dental care for this population.

Additionally, it is important to underscore the role that dental pain plays in this context. Dental pain contributed with a modest increase in dental service use among adolescents, regardless of sexual assault history. Contrary to the initial hypothesis, this association was similar in magnitude in both groups, suggesting that pain remains a relevant motivator for care‐seeking behavior even among adolescents who have experienced sexual violence. However, the relatively small magnitude of this effect indicates that other barriers to accessing dental care may persist, independent of both pain and exposure to trauma. Interestingly, Leeners *et al.* [17], considering adult women, reported that pain episodes prompted care‐seeking behaviors comparable to those without a history of abuse, although preventive attendance remained lower. In line with these findings, the present study suggests that, during adolescence, dental pain may still act as a trigger for service use, but its overall impact on utilization is limited. The coexistence of sexual trauma and dental pain may still be associated with complex emotional responses, as dental settings can trigger anxiety, distress, or re‐traumatization [20]. For these adolescents, dental treatment is not merely about alleviating physical pain but also about managing the profound emotional scars left by the trauma [12, 20].

Sex‐stratified analyses further revealed differences in the association between sexual trauma and healthcare utilization among male and female adolescents, with the associations observed primarily among girls. Although both sexes may experience increased dental anxiety and emotional distress after sexual abuse [8, 10], these burdens appear to be more evident among female adolescents, who showed slightly lower use of dental services. This pattern contrasts with normative trends, where females, even during adolescence, typically exhibit higher rates of dental service utilization [19, 28]. Interestingly, the study by Guha *et al.* [18] found that among individuals with a record of childhood sexual abuse, women had a similar pattern of dental health contacts compared to men; however, when further stratified by age of first abuse, men abused after the age of 12 had a higher frequency of using dental services than men abused before the age of 12 and both groups of women [18]. In the present study, although some differences between sexes were observed, the magnitude of these associations was modest, and no clear evidence of interaction with dental pain was identified. These findings may reflect differences in how trauma and health behaviors are expressed but should be interpreted cautiously. This underscores the importance of further research to better understand potential sex‐specific patterns and to inform tailored approaches to care [29].

In conclusion, a history of sexual assault was associated with a slightly lower use of dental services among adolescents in crude analyses, with similar magnitudes observed after adjustment. Dental pain was associated with modest increases in service use, with comparable effects among adolescents with and without a history of sexual assault. These findings suggest that, although pain remains an important driver of care‐seeking, additional barriers to accessing dental services may exist. These findings suggest that traditional dental care pathways may fail to reach this population. Therefore, integrating trauma‐informed principles into adolescent oral health care [12, 20] and expanding strategies beyond clinical settings, through school‐based programs, community outreach, and collaboration with mental health and social protection services, are essential to identify, support, and engage these vulnerable adolescents in comprehensive, equitable oral health care.

## AUTHOR CONTRIBUTIONS

**Conceptualization**: Ândrea Pires Daneris, Letícia Regina Morello Sartori, Marina da Costa Rocha, Flávio Fernando Demarco, Marcos Britto Correa, and Sarah Arangurem Karam. **Formal analysis**: Ândrea Pires Daneris, Letícia Regina Morello Sartori, Marina da Costa Rocha, and Sarah Arangurem Karam. **Investigation**: Ândrea Pires Daneris, Letícia Regina Morello Sartori, and Sarah Arangurem Karam. **Methodology**: Ândrea Pires Daneris, Letícia Regina Morello Sartori, Marina da Costa Rocha, Flávio Fernando Demarco, Marcos Britto Correa, and Sarah Arangurem Karam. **Writing—original draft**: Ândrea Pires Daneris and Letícia Regina Morello Sartori. **Writing—review and editing**: Ândrea Pires Daneris, Letícia Regina Morello Sartori, Marina da Costa Rocha, Flávio Fernando Demarco, Marcos Britto Correa, and Sarah Arangurem Karam.

## CONFLICT OF INTEREST STATEMENT

The authors declare no conflicts of interest.

## DECLARATION OF GENERATIVE AI AND AI‐ASSISTED TECHNOLOGIES IN THE WRITING PROCESS

We clarified that ChatGPT‐4o was used only to review fluency, language, spelling, and grammar, and that all AI‐assisted edits were carefully reviewed by the authors for accuracy, correctness, and scientific integrity. This tool was not used to generate content. After using the tool, the authors carefully reviewed and edited the manuscript for accuracy, correctness, and scientific integrity and take full responsibility for the content of the published article.

## Supporting information




SUPPORTING INFORMATION

## Data Availability

The data sets analyzed during the current study are publicly available on the National School Health Survey (PeNSE) website, provided by the Brazilian Institute of Geography and Statistics (IBGE), at https://www.ibge.gov.br/estatisticas/sociais/justica‐e‐seguranca/9134‐pesquisa‐nacional‐de‐saude‐do‐escolar.html?=&t=microdados.
