# Supplementary material for: Self‐reported sexual assault and dental service use among adolescents: The moderating role of dental pain
Source: Eur J Oral Sci. 2026 Jun 1;134(5):e70111. doi: 10.1111/eos.70111 (PMC13584655; doi:10.1111/eos.70111)
Supplement: Supplementary file 1 [file EOS-134-e70111-s001.docx]

**Self-Reported Sexual Assault and Dental Service Use Among Adolescents: The Moderating Role of Dental Pain**

**SUPPLEMENTARY MATERIAL**

**Table S1.** Characteristics of lifetime self-reported sexual assault among adolescents, overall and by sex: relative frequencies (%) and 95% confidence intervals (95% CI).

| **Variables/**Categories | **Total**  % (95% CI) | **Male**  % (95% CI) | **Female**  % (95% CI) |
| --- | --- | --- | --- |
| **Age at first exposure**  ≤12 years of age  13 to 15 years  16 to 17 years | 54.0 (51.8 to 56.1)  39.2 (37.1 to 41.3)  6.8 (5.8 to 8.0) | 57.6 (53.1 to 61.9)  33.2 (29.7 to 36.8)  9.2 (6.6 to 12.8) | 54.9 (51.9 to 57.8)  39.0 (36.0 to 42.0)  6.2 (5.1 to 7.4) |
| **Perpetrator ^a^**  Intimate partner (boyfriend, girlfriend, ex-partner, date, crush)  Friend  Parents (father, mother, stepfather, stepmother)  Other family members  Stranger  Another person | 25.5 (22.9 to 28.4)  18.8 (17.1 to 20.5)  12.6 (10.0 to 15.7)  20.8 (19.2 to 22.5)  18.1 (16.4 to 19.9)  15.5 (13.6 to 17.5) | 30.5 (26.2 to 35.1)  33.5 (29.9 to 37.3)  10.6 (8.0 to 13.9)  11.9 (9.6 to 14.6)  14.8 (12.2 to 17.5)  15.0 (12.6 to 17.8) | 23.8 (20.7 to 27.1)  13.5 (10.9 to 16.6)  11.9 (9.1 to 15.3)  24.6 (22.7 to 26.7)  21.0 (18.1 to 24.2)  17.0 (13.8 to 20.6) |
| *Notes: ^a^ The variable does not sum to 100% as multiple responses were allowed.* | | | |

**Table S2.** Crude and adjusted associations between adolescents’ history of sexual assault (by age at first assault) and dental service use: sensitivity analysis. Prevalence ratio (PR) and 95% confidence intervals (95% CI).

| **Variables/**Categories | **Use of dental services in the past year** | | | | | |
| --- | --- | --- | --- | --- | --- | --- |
|  | _Crude_PR (95% CI) | p-value | _Adj_PR^1^ (95% CI) | p-value | _Adj_PR^2^ (95% CI) | p-value |
| **Sexual Assault (by age at first assault)**  No  Yes, 13 to 17 years  Yes, ≤12 years of age  *Unweighted n* | 1  0.97 (0.92-1.03)  0.91 (0.87-0.96)  *123,322* | 0.397  <0.001 | 1  0.96 (0.91-1.01)  0.91 (0.87-0.96)  *120,602* | 0.179  <0.001 | 1  0.99 (0.94-1.04)  0.92 (0.87-0.98)  *101,451* | 0.862  0.011 |
| ***Sex stratified*** |  |  |  |  |  |  |
| ***Male*** |  |  |  |  |  |  |
| **Sexual Assault (by age at first assault)**  No  Yes, 13 to 17 years  Yes, ≤12 years of age  *Unweighted n* | 1  1.03 (0.92-1.16)  0.91 (0.83-0.99)  *60,379* | 0.543  0.040 | 1  1.04 (0.93-1.17)  0.92 (0.84-1.00)  *59,140* | 0.433  0.066 | 1  1.09 (0.94-1.19)  0.92 (0.84-1.01)  *48,640* | 0.309  0.100 |
| ***Female*** |  |  |  |  |  |  |
| **Sexual Assault (by age at first assault)**  No  Yes, 13 to 17 years  Yes, ≤12 years of age  *Unweighted n* | 1  0.94 (0.88-1.00)  0.90 (0.85-0.95)  *62,657* | 0.056  <0.001 | 1  0.93 (0.87-0.99)  0.90 (0.85-0.96)  *61,462* | 0.030  0.001 | 1  0.95 (0.89-1.01)  0.93 (0.87-0.98)  *52,811* | 0.154  0.019 |
| *Notes. Analytical sample sizes (unweighted n) vary across models due to item-specific missing data and complete case analysis. Weighted estimates account for the complex survey design. Adjusted prevalence ratios (_Adj_PR^1^) for sex, age, and skin color. Adjusted prevalence ratios (_Adj_PR^2^) for Brazilian region, school area, school administrative structure, sex, age, skin color, maternal schooling, living with parents, and physical aggression by parents or guardians in the past year. In sex-stratified models, the sex variable was not included in the adjustment as stratification already accounts for its confounding role.* | | | | | | |

**Table S3.** Sensitivity analysis of adjusted associations between adolescents’ history of sexual assault and use of dental services in the past year: impact of missing maternal schooling data. Prevalence ratio (PR) and 95% confidence intervals (95% CI).

| **Variables/**Categories | **Use of dental services in the past year** | |
| --- | --- | --- |
|  | _Adj_PR (95% CI) | *p-value* |
| ***Model 1*** |  |  |
| **Sexual Assault (101,451)**  No  Yes | 1  0.96 (0.92-1.00) | 0.051 |
| *Female* |  |  |
| Sexual Assault (52,811)  No  Yes | 1  0.94 (0.89-0.98) | 0.010 |
| ***Model 2*** |  |  |
| **Sexual Assault (120,232)**  No  Yes | 1  0.94 (0.91-0.98) | 0.006 |
| *Female* |  |  |
| Sexual Assault (61,317)  No  Yes | 1  0.93 (0.89-0.97) | 0.001 |
| *Notes. Model 1: Adjusted prevalence ratios (PR) for the Brazilian region, school area, school administrative structure, sex, age, skin color, maternal schooling, living with parents, and physical aggression by parents or guardians in the past year. Model 2 adjusted for all previous variables except maternal schooling.* | | |

**Table S4.** Dental service use according to response category for lifetime sexual assault, including item nonresponse and system-missing cases: relative frequencies (%) and 95% confidence intervals (95% CI).

| **Variables/**Categories | **Total** (%) | **Use of dental service in the past year** | | ***p-value*** |
| --- | --- | --- | --- | --- |
|  |  | No  % (95% CI) | Yes  % (95% CI) |  |
| **Sexual assault (124,106)**  No  Yes  System missing  No response | 93.1  6.1  0.2  0.6 | 33.0 (32.3 to 33.8)  36.8 (34.5 to 39.2)  37.6 (25.6 to 51.3)  33.5 (26.4 to 41.5) | 67.0 (66.2 to 67.7)  63.2 (60.8 to 65.5)  62.4 (48.7 to 74.4)  66.5 (58.5 to 73.6) | 0.024 |
| **Total (124,106)** | - | 33.3 (32.6 to 33.9) | 66.7 (66.1 to 67.4) | - |
| *Notes. Weighted percentages account for the survey design. System missing refers to questionnaire abandonment; No response refers to item nonresponse.* | | | | |

**Table S5.** Crude and adjusted associations between adolescents’ history of sexual assault (including item nonresponse and system-missing cases and a worst-case scenario) and dental service use: sensitivity analysis. Prevalence ratio (PR) and 95% confidence intervals (95% CI).

| **Variables/**Categories | **Use of dental services in the past year** | | | | | |
| --- | --- | --- | --- | --- | --- | --- |
|  | _Crude_PR (95% CI) | p-value | _Adj_PR^1^ (95% CI) | p-value | _Adj_PR^2^ (95% CI) | p-value |
| **Sexual Assault (including missing)**  No  Yes  System missing  No response  *Unweighted n* | 1  0.94 (0.91-0.98)  0.93 (0.75-1.15)  0.99 (0.88-1.11)  *124,106* | 0.003  0.517  0.906 | 1  0.93 (0.90-0.97)  0.96 (0.77-1.19)  1.01 (0.89-1.14)  *121,368* | 0.001  0.726  0.808 | 1  0.95 (0.92-0.99)  1.13 (0.78-1.63)  1.01 (0.84-1.20)  *101,726* | 0.050  0.491  0.895 |
| **Sexual Assault (worst-case scenario)**  No  Yes  *Unweighted n* | 1  0.94 (0.91-0.98)  *124,106* | 0.007 | 1  0.94 (0.91-0.98)  *121,368* | 0.003 | 1  0.96 (0.92-1.00)  *101,726* | 0.073 |
| *Notes. Analytical sample sizes (unweighted n) vary across models due to item-specific missing data and complete case analysis. Weighted estimates account for the complex survey design. Adjusted prevalence ratios (_Adj_PR^1^) for sex, age, and skin color. Adjusted prevalence ratios (_Adj_PR^2^) for Brazilian region, school area, school administrative structure, sex, age, skin color, maternal schooling, living with parents, and physical aggression by parents or guardians in the past year.*  *System missing refers to questionnaire abandonment; No response refers to item nonresponse. In the worst-case scenario, item nonresponse and system-missing cases were conservatively classified as having experienced sexual assault, assuming that all missing responses corresponded to affirmative exposure.* | | | | | | |
